# Lipoprotein(a) as a Risk Factor for Recurrent Acute Coronary Syndrome and Unplanned Revascularization in Fractional Flow Reserve-Negative Lesions

**DOI:** 10.31083/RCM47169

**Published:** 2026-03-20

**Authors:** Yingying Zhao, Jiayi Luo, Kai Xu, Yang Li, Yaling Han

**Affiliations:** ^1^Department of Cardiology, Laboratory of Frigid Zone Cardiovascular Disease, General Hospital of the PLA Northern Theater Command, 110016 Shenyang, Liaoning, China

**Keywords:** acute coronary syndrome, percutaneous coronary intervention, fractional flow reserve, lipoprotein(a), revascularization

## Abstract

**Background:**

This study aimed to explore the association between serum lipoprotein(a) [Lp(a)] levels and recurrent acute coronary syndrome (ACS) and revascularization of target lesions in patients with ACS who showed no functional ischemia on fractional flow reserve (FFR) testing during coronary angiography (CAG).

**Methods:**

The retrospective observational study was conducted at the General Hospital of Northern Theater Command and included 513 patients with new ACS recruited from 23 February 2016 to 6 November 2023 and followed up. These patients underwent CAG examination and were found to have at least one coronary artery with moderate or greater stenosis, and also underwent FFR measurement with FFR value >0.80. Patients experienced recurrent ACS and underwent unplanned revascularization were defined as the revascularization group, while patients did not experience recurrent ACS and undergo unplanned revascularization were assigned to the no revascularization group. The study employed propensity score matching (PSM) and receiver operating characteristic (ROC) curve analysis to evaluate the correlation between serum Lp(a) and recurrent ACS and unplanned revascularization in target lesion with FFR value >0.80.

**Results:**

Serum Lp(a) levels were higher in female patients. There were no statistically significant differences in the basic clinical characteristics, medication use, laboratory test results or ejection fraction values between the two groups. During a average follow-up of 6.5 years, 119 patients (23.2%) experienced recurrent ACS and unplanned revascularization in the target lesion. The level of serum Lp(a) in the patients that underwent unplanned revascularization was significantly higher than in the group that did not undergo repeated revascularization (65.80 mmol/L vs. 60.57 mmol/L, *p* = 0.034).

**Conclusion:**

Serum Lp(a) is an independent risk factor for recurrent ACS and unplanned revascularization in patients with ACS and FFR negative plaque.

## 1. Introduction

Acute coronary syndrome (ACS) is mostly caused by the erosion or rupture of unstable atherosclerotic plaques, leading to acute thrombus formation in the coronary arteries and resulting in acute myocardial ischemia, which manifests as a clinical syndrome [1]. The primary treatment modality continues to be percutaneous coronaryintervention (PCI) as the preferred initial approach, and coronary angiography (CAG) remains the established standard for evaluating the severity of ischemia in coronary artery disease [2, 3]. With the in-depth research conducted by domestic and international scholars on coronary artery function and physiology, it has gradually been recognized that CAG, which only provides a subjective visual assessment of coronary artery stenosis from an anatomical perspective, cannot accurately determine whether a patient has clinically significant functional ischemia, resulting in patients without functional ischemia being unable to truly benefit from revascularization [4]. Pijls *et al*. [5] compared fractional flow reserve (FFR) with coronary angiography, exercise stress myocardial scintigraphy, and pharmacologic stress echocardiography, confirming that FFR can provide hemodynamic information about vascular stenotic lesions and objectively evaluate the relevant lesions associated with myocardial ischemia [6, 7]. The measurement of FFR can facilitate the determination of the reversibility of coronary artery stenosis from hemodynamic and physiological perspectives. Furthermore, it provides substantial evidence to inform the decision regarding the necessity of revascularization of the affected segment [8, 9].

In recent years, cardiovascular-related deaths have remained the leading cause of death among urban and rural residents in China. It is widely acknowledged that age, smoking, hypertension, diabetes, dyslipidaemia, and obesity are established risk factors for coronary heart disease. Consequently, the primary focus of coronary heart disease prevention and control has historically been on implementing early interventions. However, even when traditional risk factors are adequately controlled, some patients do not demonstrate complete improvement in their clinical prognosis [10]. This suggests the possibility of additional risk factors in coronary heart disease patients. Serum Lp(a) is a distinct form of lipoprotein particle that was first identified by Kare Berg over 60 years ago [11]. It is a class of lipoproteins that are synthesized independently by the liver and are incapable of being converted into other lipoproteins [12, 13]. Its concentration is genetically determined and remains relatively stable, largely unaffected by gender, age, weight, and most clinically used lipid-lowering drugs [14, 15]. In recent years, multidimensional studies have consistently indicated that serum Lp(a) is an independent risk factor for atherosclerotic cardiovascular disease (ASCVD) independent of low-density lipoprotein cholesterol (LDL-C) [16, 17, 18]. Recent reports have demonstrated a significant correlation between elevated levels of LDL-C and serum Lp(a) and the vulnerability of newly formed atherosclerotic plaques [19, 20]. However, the aetiological mechanism of atherosclerosis has not yet been fully elucidated, and it may be related to its role in promoting thrombosis, triggering vascular inflammatory responses, and accelerating the progression of atherosclerosis [21, 22, 23].

Despite the growing focus on clinical research related to serum Lp(a) in recent years, there remains a significant gap in the availability of related research studies. In order to provide further clarification regarding its clinical value as a biomarker, this paper assesses its predictive value for the occurrence and risk of ACS by exploring the correlation between serum Lp(a) levels and the severity of coronary artery lesions in ACS patients with negative FFR (>0.8).

## 2. Materials and Methods

### 2.1 Data Collection

The present study constitutes a single-center retrospective study, which selected patients diagnosed with ACS at our center between 2016 and 2023 who underwent CAG and FFR examinations during their hospitalization. Using the Digital Subtraction Angiography (DSA) system to acquire images, the results of coronary angiography (CAG) were visually interpreted by two experienced interventional cardiologists. The CAG examination revealed moderate or greater stenosis in at least one coronary artery. Exclusion criteria: (1) Concurrent severe liver or kidney dysfunction, severe infection, thyroid dysfunction, tumours, hypertrophic cardiomyopathy, or aortic valve stenosis; (2) Use of steroids or chemotherapy within the past 3 months; (3) Use of nicotinic acid, proprotein convertase subtilisin/kexin type 9 (PCSK9) inhibitors, or other drugs affecting serum Lp(a) within the past month. Primary endpoint was any revascularization during follow-up.

A review of the hospital information (HIS) system was undertaken in order to obtain case data that met the requisite requirements. Primary ebdpoint was any revascularization.

### 2.2 Coronary Angiography and Flow Reserve Measurement

In order to measure FFR in the coronary artery lesion segment of a patient, it is necessary to position the pressure guidewire of the FFR system distal to the stenotic lesion, with the proviso that the tip of the pressure guidewire remains centered in the vascular lumen. The administration of adenosine intravenously is required to achieve maximum vasodilation. The pressure distal to the coronary artery stenosis (Pd) is measured via the pressure catheter, and the pressure proximal to the coronary artery (Pa) is measured via the guide catheter. This results in the following formula for fractional flow reserve (FFR): FFR = Pd/Pa. FFR ≤0.80 is the gold standard for diagnosing functional coronary artery ischemia. In this study, all patients exhibited an initial FFR value greater than 0.80. Patients were subjected to regular telephone follow-ups and categorized into two distinct groups, namely the repeat revascularization group and the non-repeat revascularization group, based on whether they underwent any revascularization.

### 2.3 Lipoprotein(a) Analysis

Laboratory parameters encompassing total cholesterol (TC), triglyceride (TG), high-density lipoprotein cholesterol (HDL-C), LDL-C, and Lp(a) assays (Plasma concentrations of the aforementioned indicators were measured in 3 mL of venous blood drawn on an empty stomach the morning after admission). The blood sample was permitted to coagulate for a period of one hour prior to analysis using a Beckman Coulter AU5800 biochemical analyser. TC was measured using the Cholesterol Oxidase-Peroxidase-4-Aminoantipyrine Method, expressed in mmol/L. The TG levels were measured using the Glycerol-3-Phosphate Oxidase-Peroxidase-4-Aminoantipyrine Method, with the results expressed in mmol/L. The determination of HDL-C and LDL-C was conducted through the utilisation of direct enzymatic assays, with the resultant values expressed in mmol/L. Serum Lp(a) was measured by means of an immunoturbidimetric assay, with results expressed in mmol/L.

### 2.4 Statistical Analysis

Continuous variables are expressed as mean ± standard deviation or median (interquartile range). Intergroup comparisons are performed using Student’s *t*-test (for normally distributed data) or Mann-Whitney U test (for non-normally distributed data). Categorical variables are expressed as counts and percentages. The statistical analysis of intergroup comparisons is typically conducted through the utilization of either the Chi-Square test or the Fisher’s exact probability test, depending on the nature of the data and the research question being addressed. In order to identify indicators capable of predicting high-risk coronary artery plaques, univariate logistic regression analysis was used to examine the association between each indicator and repeat revascularization. Receiver operating characteristic (ROC) curves were utilized to analyze the correlation between each indicator and repeat revascularization. All statistical analyses were performed using SPSS (26.0; IBM Corporation, Armonk, NY, USA) and R language (Version 4.5.0; R Foundation for Statistical Computing, Vienna, Austria). A two-sided *p*-value of less than 0.05 was considered to be statistically significant Secondly, propensity score matching was implemented using an extension to the R language. The vascular reconstruction group and the non-vascular reconstruction group were designated as the dependent variables, respectively. Variables that showed significant differences between the two groups prior to propensity score matching (PSM) were used as covariates. The two datasets were matched using a 1:1 nearest neighbour matching method. Following multiple iterations, the calibration values for both groups were ultimately determined to be 0.02. The aforementioned statistical methods were then applied to compare the matched datasets. Variables showing significant differences between the groups had *p*-values greater than 0.05 and absolute standardised differences of less than 20%. This indicates satisfactory balance between the matched cohorts.

## 3. Results

### 3.1 Comparison of General Characteristics

**Fig. 1.**
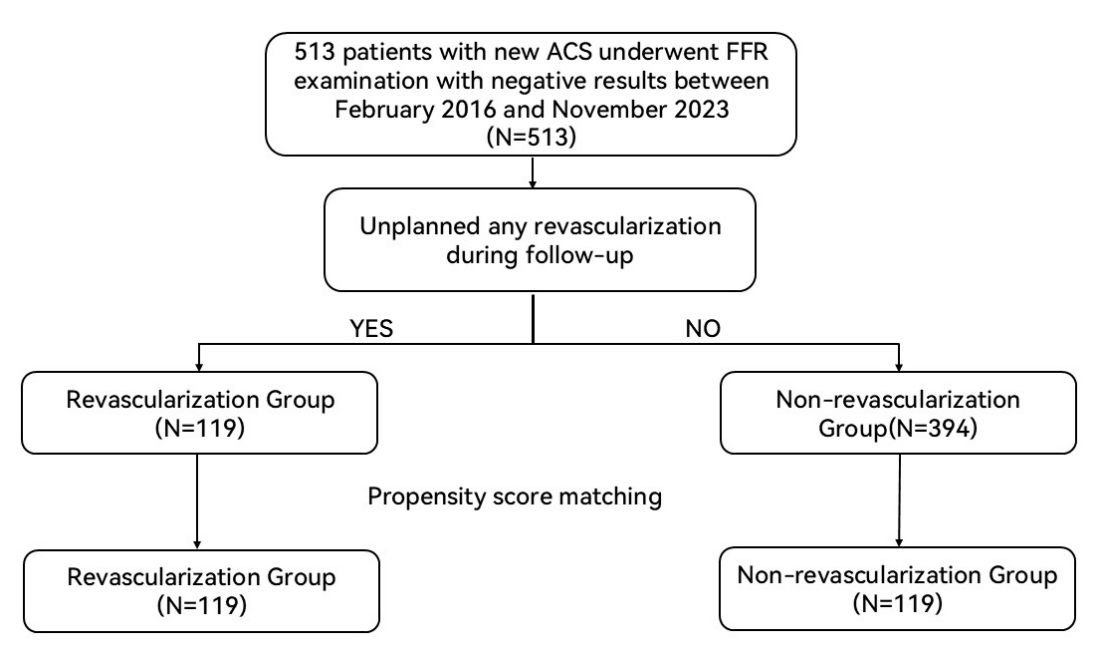
**Study flow**. ACS, acute coronary syndrome; FFR, fractional flow reserve.

A total of 513 patients with new ACS were recruited from 23 February 2016 to 6 November 2023 and underwent follow-up. The patients with ST-elevation myocardial infarction (STEMI) who were included in this study presented within 7 to 30 days of symptom onset, with target coronary artery stenosis ranging between 50% and 70%. All patients underwent CAG examination and FFR measurement. Patients who experienced recurrent ACS and underwent unplanned revascularization were defined as the revascularization group (n = 119). Those who did not experience recurrent ACS and undergo unplanned revascularization were assigned to the no revascularization group (n = 394). Subsequently, propensity score matching was performed at a 1:1 ratio to minimize confounding factors. Following the matching process, the revascularization group and the no revascularization group each comprised 119 patients (Fig. 1).

**Fig. 2.**
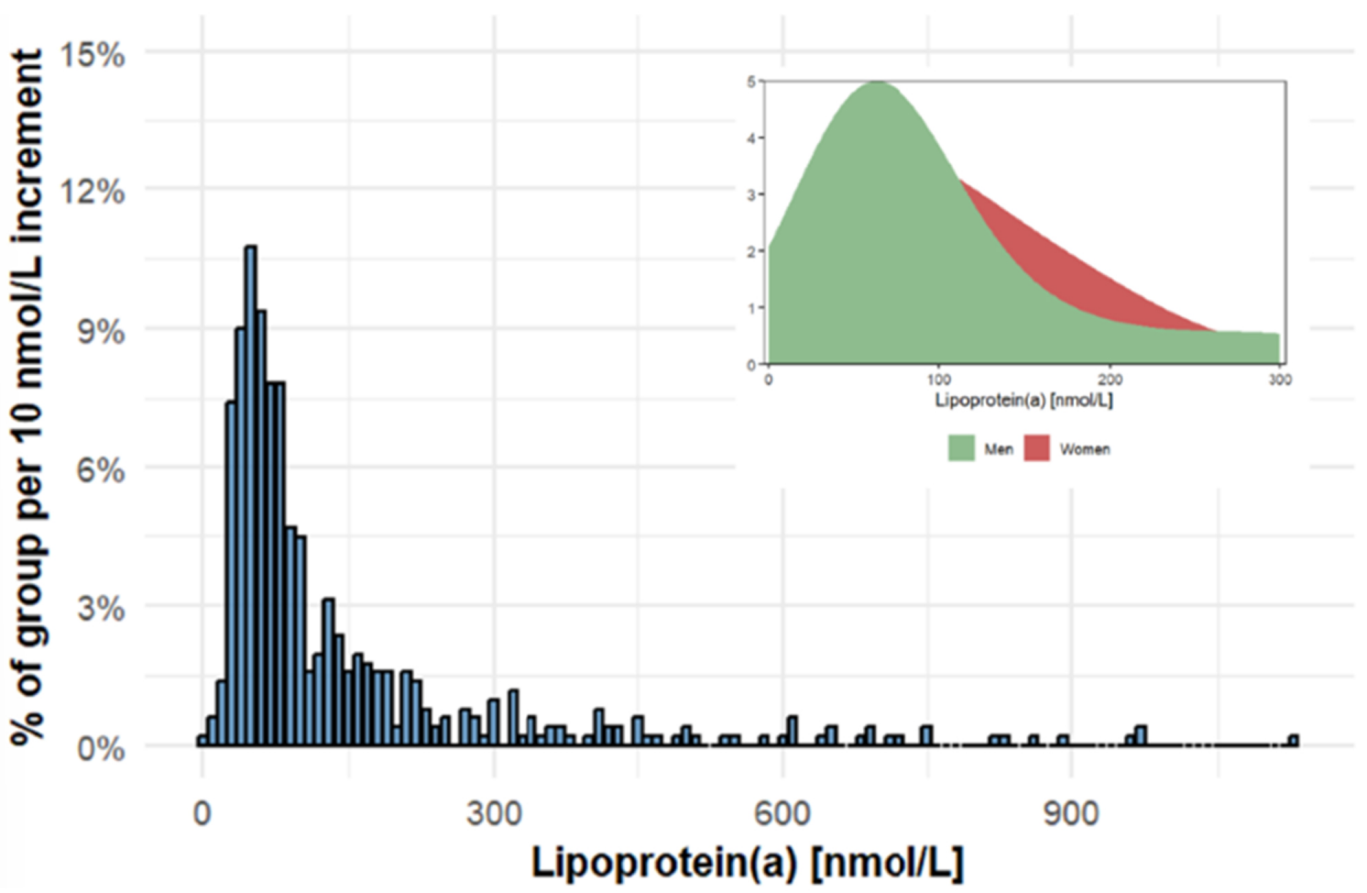
**Distribution of Lipoprotein(a) in the study population**.

The baseline characteristics of the patients are set out in Table 1. The majority of subjects exhibited lower concentrations of serum Lp(a), whilst only a minority demonstrated elevated levels of serum Lp(a). Conversely, there are discernible disparities in the distribution of serum Lp(a) between the sexes. It is evident that a greater proportion of the female population may exhibit elevated levels of serum Lp(a) concentrations (Fig. 2). A comparison of patients’ characteristics in the non-revascularization group and those in the revascularization group revealed no significant differences in terms of age, sex, body mass index (BMI), and the proportion of patients with hypertension and diabetes, and current smoking prevalence, as well as medication use. In the serological related indicators of the two groups of patients, no statistically significant difference was observed in the comparison of total cholesterol, LDL-C, HDL-C and triglyceride. However, a significant difference in serum Lp(a) levels was observed between the two groups [65.80 (50.25–85.91) mmol/L vs 60.57 (50.25–93.38) mmol/L, *p* = 0.034].

**Table 1.** **Patient characteristics**.

Characteristic		Before Matching			After Matching		
		Revascularization (n = 119)	Non-Revascularization (n = 394)	*p* Value	Revascularization (n = 119)	Non-Revascularization (n = 119)	*p* Value
Demographic Data							
	Age—yr	69 (66–73)	69 (64–75)	0.901	69 (66–73)	70 (64–75)	0.627
	Male sex—no. (%)	83 (69.70)	235 (59.60)	0.060	83 (69.70)	53 (44.50)	0.714
	Hypertension—no. (%)	69 (58.00)	245 (62.20)	0.596	69 (58.0)	53 (44.50)	0.617
	Diabetes mellitus—no. (%)	33 (27.70)	140 (35.50)	0.142	33 (27.70)	30 (25.30)	0.714
	BMI—kg/m^2^	23.2 (21.00–25.30)	23.8 (21.70–25.60)	0.142	23.2 (21.00–25.30)	23.11 (21.00–25.10)	0.318
	Current smoker—no. (%)	17 (14.30)	87 (22.10)	0.662	17 (14.30)	37 (27.70)	0.512
	Previous stroke—no. (%)	21 (17.65)	38 (14.60)	0.106	21 (25.90)	19 (15.90)	0.699
	Mean follow-up time—yr	6.50 ± 1.30	6.30 ± 1.40	0.135	6.50 ± 1.30	6.30 ± 1.30	0.211
Laboratory data							
	LDL-C—mmol/L	1.97 (1.72–2.20)	2.03 (1.65–2.37)	0.287	1.97 (1.72–2.20)	1.96 (1.74–2.19)	0.964
	HDL-C—mmol/L	1.03 (0.86–1.29)	1.06 (0.88–1.33)	0.251	1.03 (0.86–1.29)	1.08 (0.84–1.33)	0.183
	Lp(a)—mmol/L	65.80 (50.25–85.91)	92.30 (52.51–193.5)	<0.001	65.80 (50.25–85.91)	60.57 (50.25–93.38)	0.034
	TC—mmol/L	4.02 (3.16–5.07)	3.58 (2.94–4.30)	0.002	4.02 (3.16–5.07)	3.54 (2.92–4.76)	0.616
	TG—mmol/L	1.28 (0.96–1.72)	2.00 (1.05–2.67)	<0.001	1.28 (0.96–1.72)	2.01 (1.04–2.66)	0.674
	Serum creatinine level—µmol/L	72.30 (61.15–86.20)	66.00 (56.52–76.63)	<0.001	72.30 (61.15–86.20)	71.70 (60.90–84.70)	0.633
	HGB—g/L	152.00 (141.50–157.00)	133.00 (122.00–147.00)	<0.001	152.00 (141.50–157.00)	146.0 (136.50–154.00)	0.924
	Platelet—10^9^/L	238.00 (195.50–277.50)	219.75 (183.25–262.50)	0.006	238.00 (195.50–277.50)	236.0 (194.50–278.50)	0.572
	LVEF—%	63 (62–64)	62 (61–63)	<0.001	63 (62–64)	63 (62–63)	0.437
Concomitant Medications—no. (%)							
	Aspirin	117 (98.30)	374 (94.90)	0.024	117 (98.30)	74 (98.70)	0.604
	ADP inhibitor	69 (57.90)	227 (57.60)	0.056	69 (57.90)	48 (48.70)	0.994
	Statin	116 (97.50)	390 (99.00)	0.036	116 (97.50)	74 (98.70)	0.604
	ACEI or ARB	65 (54.60)	189 (48.00)	0.243	65 (54.60)	42 (56.00)	0.998
	Beta-blocker	77 (64.70)	231 (58.60)	0.280	77 (64.70)	46 (61.30)	0.494
	Calcium-channel blocker	22 (18.50)	84 (21.30)	0.589	22 (18.50)	11 (14.70)	0.824

LDL-C, low-density lipoprotein cholesterol; BMI, body mass index; HDL-C, high-density lipoprotein cholesterol; Lp(a), lipoprotein(a); TC, total cholesterol; TG, triglyceride; HGB, hemoglobin; LVEF, left ventricular ejection fraction; ADP, adenosine diphosphate; ACEI, angiotensin-converting enzyme inhibitor; ARB, angiotensin receptor blocker.

The angiographic characteristics of the patients are summarized in Table 2. A comparison between the non-revascularization and revascularization groups showed no significant differences in FFR values, percentage of diameter stenosis, arterial access types, number of target vessels per patient, or target vessel distribution.

**Table 2.** **Angiography characteristics**.

Characteristic		Revascularization (n = 119)	Non-Revascularization (n = 119)	*p* Value
Arterial access—no. (%)				0.886
	Transradial	114 (95.80)	105 (88.24)	
	Transfemoral	5 (4.20)	14 (11.76)	
Target vessels per patient—no. (%)				0.545
	1	100 (84.00)	108 (90.80)	
	2	19 (16.00)	11 (9.20)	
Target vessel—no./total no. (%)				0.642
	LAD	64/122 (52.46)	83/119 (69.75)	
	LCX	40/122 (32.79)	13/119 (10.92)	
	RCA	18/122 (14.75)	23/119 (19.32)	
Diameter stenosis—%		61.60 ± 5.70	61.80 ± 4.60	0.112
FFR		0.86 (0.82–0.89)	0.85 (0.83–0.88)	0.591

FFR, fractional flow reserve; LAD, left anterior descending artery; LCX, left circumflex artery; RCA, right coronary artery.

### 3.2 Diagnostic Efficacy of Serum Lp(a) in Re-Revascularization

**Fig. 3.**
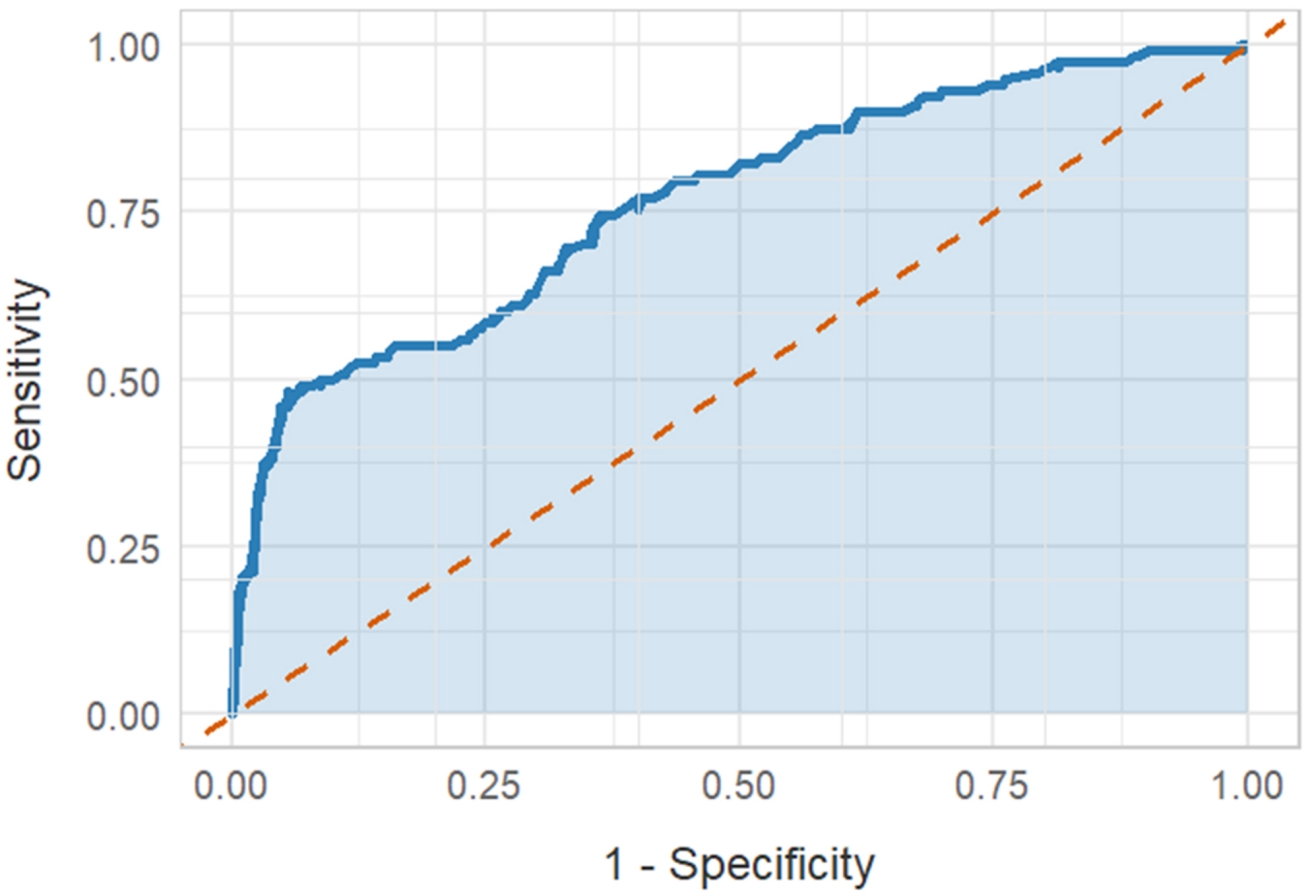
**ROC analysis**. ROC, receiver operating characteristic.

In univariate logistic regression analysis, serum Lp(a) (OR: 1.008, 1.006–1.010, *p* < 0.001) was identified as an independent predictor of re-revascularization. This study evaluated the efficacy of serum Lp(a) in predicting recurrent ACS and unplanned revascularization procedures through ROC curve analysis. The ROC curve (Fig. 3) indicates that the area under the curve (AUC) for serum Lp(a) is 0.869, which suggests a favourable predictive value. The optimal cut-off value of 91.245 was determined using the maximum Yorden index method. At this threshold, serum Lp(a) showed a sensitivity of 81.4%, a specificity of 93.3% and a Yorden index of 0.747. Furthermore, the positive predictive value (PPV) of an serum Lp(a) diagnosis was 83.05% and the negative predictive value (NPV) was 83.19%. In summary, serum Lp(a) demonstrates high clinical utility in predicting recurrent ACS and unplanned revascularization with 91.245 as the optimal cut-off value.

## 4. Discussion

The conclusions of this study are as follows. Firstly, the serum Lp(a) levels in the revascularization group were significantly higher than those in the non-revascularization group. Secondly, serum Lp(a) has been identified as an independent risk factor for predicting re-revascularization.

Recent studies have demonstrated that delaying PCI in patients with FFR >0.8 does not increase cardiovascular event rates. This study similarly found that among the 513 patients enrolled, after an average follow-up of 6.5 years, 119 patients did indeed undergo target vessel revascularization. Despite receiving standardised treatment and demonstrating relatively stable risk factor control during follow-up, these patients still experienced target vessel revascularization failure. However, the specific reasons and the patients prone to target vessel revascularization remain unclear. This uncertainty underscores the significance of this study.

In recent years, with the continuous deepening of research on ACS, significant progress has also been made in related research on cardiovascular disease markers. While it is acknowledged that different markers may reflect various stages of the ACS disease process to a certain extent, it is important to recognize the limitations of these markers in clinical application. Neither imaging tests such as CAG and intravascular ultrasound nor clinical information obtained through non-invasive means such as troponin, cardiac ultrasound, and electrocardiogram can detect unstable plaque rupture and thrombus formation at an early stage. PCI and lipid-lowering therapy have been demonstrated to enhance the prognosis of ACS patients; however, the mortality rate and risk of rehospitalization among ACS patients remain high. LDL-C is widely acknowledged as a significant risk factor for the development of coronary heart disease. Nevertheless, a proportion of patients have been observed to experience cardiovascular events despite achieving target LDL-C levels, as indicated by the findings of certain studies [24]. Concurrently, the role of serum Lp(a) in predicting the risk of coronary heart disease is also receiving increasing attention. This is due to the fact that serum Lp(a) concentrations are relatively stable in individuals and are primarily influenced by genetic factors, thus making it a reliable indicator for predicting the severity of coronary artery stenosis.

In order to predict the occurrence of cardiovascular events at an early stage, serum Lp(a) has become a highly regarded lipid-lowering target in recent years. However, the precise mechanism by which elevated serum Lp(a) levels lead to cardiovascular disease remains to be elucidated. Consequently, it is now particularly urgent and necessary to explore how to reduce serum Lp(a) levels through drug intervention, with the objective of mitigating the risk of cardiovascular events, providing patients with more effective treatment strategies, and enhancing their clinical outcomes. From a pathophysiological perspective, serum Lp(a) is an important component of blood lipids, primarily composed of low-density lipoprotein particles rich in cholesterol. These particles are formed by the covalent bonding of apo(A) and apo(B) via disulphide bonds [25, 26]. Serum Lp(a) has been demonstrated to increase the risk of repeat revascularization in ACS patients through multiple mechanisms [27, 28]. It has been hypothesized by some researchers that serum Lp(a) possesses a comparable structural configuration to plasminogen, yet it is deficient in protease functionality [28]. This structural similarity may enable serum Lp(a) to interfere with the normal function of plasminogen through a mechanism known as ‘molecular mimicry’, thereby inhibiting the fibrinolysis process and ultimately increasing the risk of thrombosis. Serum Lp(a) may cause endothelial cell dysfunction, which leads to a significant increase in tissue factor secretion. Tissue factor can further stimulate the extrinsic coagulation pathway, thereby inhibiting the natural anticoagulant function of the endothelium [29]. Concurrently, the serine domain on the surface of serum Lp(a) has the capacity to bind to tissue factor pathway inhibitor (TFPI), thereby inhibiting the activity of TFPI and indirectly promoting thrombus formation [29]. A complex interaction exists between serum Lp(a) and platelets, with both promoting and inhibiting platelet aggregation [29, 30]. However, the conclusions of current clinical research remain inconsistent, and further investigation is required into the mechanisms of action between serum Lp(a) and various platelet receptors. The primary lipid-lowering therapies currently available, such as statins, fibrates and cholesterol absorption inhibitors, have virtually no effect on serum Lp(a) levels [31]. PCSK9 inhibitors, which have been demonstrated to be effective agents for lowering LDL-C, have been shown to reduce serum Lp(a) levels by 20–30% through the inhibition of serum Lp(a) synthesis [32]. However, for patients with elevated baseline serum Lp(a) levels, the reduction achieved following PCSK9 inhibitor therapy remains far from sufficient. Therefore, drugs that reduce serum Lp(a) warrant vigorous research efforts to provide a theoretical foundation for their more effective clinical application. reduce serum Lp(a) levels, and commonly used lipid-lowering drugs cannot reliably reduce serum Lp(a) levels. In comparison with patients who did not undergo revascularization, ACS patients who underwent PCI received conventional intensive drug therapy. Furthermore, baseline high serum Lp(a) levels exacerbated thrombosis, worsened coronary artery stenosis, and increased the risk of recurrent ischemic events.

The formation and progression of atherosclerotic plaques are currently the most common causes of myocardial ischemia. The question of whether coronary artery stenosis directly causes myocardial ischemia and whether its severity directly affects patient prognosis, leading to different clinical outcomes, remains unresolved [33]. In cases where the degree of coronary artery stenosis is mild and has not resulted in severe myocardial ischemia, the administration of pharmaceutical therapy may yield favorable outcomes. In the absence of such measures, revascularization is regarded as the optimal therapeutic intervention for patients. FFR is a functional indicator that evaluates the degree of narrowing of the coronary artery by measuring the pressure inside the coronary artery. The purpose of this investigation is to determine whether the diseased coronary artery is associated with myocardial ischemia. In recent years, FFR has gradually become an important reference indicator for clinicians to assess the anatomical and physiological functions of narrowed coronary arteries. FFR results can demonstrate hemodynamic information at the site of vascular stenosis and objectively evaluate the functional changes caused by coronary artery stenosis. However, while FFR measurements enable the precise formulation of treatment measures, the relatively lengthy measurement process extends the operator’s exposure to radiation and some patients are unable to tolerate the adverse reactions of vasodilators.

In relation to clinical practice, the findings of this study are as follows. Firstly, when assessing treatment options for patients with ACS, clinicians should not rely solely on CAG and FFR measurements as criteria for determining PCI treatment; they should also pay attention to test indicators such as serum Lp(a). For patients with elevated serum Lp(a) levels who have not undergone revascularization therapy, a more proactive approach to follow-up and timely intervention is recommended. This may include shortening follow-up intervals, intensifying symptom monitoring, and considering enhanced lipid-lowering and anticoagulant therapy regimens to slow the progression of atherosclerosis. Secondly, elevated serum Lp(a) is clearly associated with genetic predisposition, but through multidimensional risk assessment, strict control of LDL-C levels and other risk factors, targeted intervention for thrombosis risk, and active research and development of new targeted drugs to lower serum Lp(a), it is still possible to effectively reduce overall cardiovascular risk [34, 35]. It is imperative that clinicians do not prioritize solely the alleviation of vascular stenosis; rather, they should devise treatment strategies that optimize future benefits for patients and enhance their clinical outcomes.

It is noteworthy that no similar phenomenon was observed in the PCI treatment group guided by FFR in this study. The hypothesis that this is related to the fact that PCI mechanically opens coronary artery stenosis, which may induce neointimal hyperplasia to form a protective ‘new cap’ and thereby stabilise the underlying plaque, potentially reducing the risk of subsequent events, remains to be further verified [36, 37]. The extent to which PCI treatment can offset the adverse effects of high serum Lp(a) remains to be demonstrated by large-scale clinical studies. Nevertheless, it is important to note that patients who undergo PCI should not disregard serum Lp(a) postoperatively. For patients with high serum Lp(a) levels who have undergone PCI, close monitoring and control of their serum Lp(a) levels in the future is imperative in order to reduce the risk of complications such as stent thrombosis and restenosis. The present study revealed that there were no significant differences between the two groups of patients in terms of blood lipid indicators other than serum Lp(a). This outcome may be attributable to the fact that such indicators are more influenced by acquired factors (e.g., diet, obesity, and metabolic syndrome) and typically increase gradually after middle age [38]. The level of serum Lp(a) is genetically determined and not influenced by lifestyle factors such as diet or exercise. Conventional medications are also ineffective in reducing the symptoms. Individuals with high serum Lp(a) levels may be in a state of ‘continuous exposure’ to atherosclerosis from birth. However, the question of whether serum Lp(a) is more sensitive or leads to coronary artery atherosclerosis earlier than LDL-C remains inconclusive at present.

### Limitations

The present study is subject to certain limitations. Firstly, it is a single-centre retrospective study using a non-randomised design. Secondly, the serum Lp(a) levels of the patients in the study were relatively low. Further research is required on patients with high serum Lp(a) levels to clarify the relationship between serum Lp(a) and myocardial ischemia. Thirdly, the process of FFR testing necessitates that the coronary artery be in a state of maximum congestion. In patients with diabetes and microvascular lesions or acute ST-segment elevation myocardial infarction, microvascular obstruction has been shown to reduce the dilatory effect of drugs, resulting in false negative FFR values [39]. In such circumstances, FFR measurements are unable to accurately reflect the actual physiological state of the coronary arteries. Furthermore, the study did not encompass additional clinical outcome indicators, such as cardiovascular death, stroke, and myocardial infarction, which hinders a comprehensive evaluation of the impact of serum Lp(a) on the long-term prognosis of ACS patients. Fourthly, FFR only indicates the functional status of the diseased vessel; it does not reveal the histological characteristics of the coronary lesion itself or the instability of the plaque. However, if it were combined with intravascular ultrasound and/or optical coherence tomography, it would provide insights from anatomical, haemodynamic and functional perspectives, offering more comprehensive guidance for PCI, clinical management and prognosis. This approach would improve myocardial perfusion, reduce the risk of long-term adverse cardiovascular events, and enhance clinical outcomes.

## 5. Conclusion

Our data show that for the same level of serum serum Lp(a), more women than men are at risk of a recurrent ASCVD event due to high serum Lp(a) in the patients with ACS and negative FFR results. Higher serum Lp(a) levels were associated with increasing risk of a recurrent ACS event and unplanned revascularization. This finding reveals the predictive role of serum Lp(a) in the treatment prognosis of ACS patients and provides new directions for clinicians in cardiovascular disease risk stratification and intervention. Standardized measurement and early screening can facilitate the optimization of prevention strategies for high-risk populations.

## Availability of Data and Materials

The data that support the findings of this study are available from the corresponding author, upon reasonable request.
